# The Determinants of Brownfields Redevelopment in England

**DOI:** 10.1007/s10640-015-9985-y

**Published:** 2016-01-07

**Authors:** Alberto Longo, Danny Campbell

**Affiliations:** 1 Gibson Institute for Land, Food and Environment, Institute for Global Food Security, School of Biological Sciences, UKCRC Centre of Excellence for Public Health, Queen’s University Belfast, Belfast, UK; 2 Basque Centre for Climate Change (BC3), 48008 Bilbao, Spain; 3 Economics Division, Stirling Management School, University of Stirling, Stirling, UK

**Keywords:** Brownfields, GIS, Revealed preferences, Binary choice model, Spatial autocorrelation, Spatial probit latent class model

## Abstract

This paper uses discrete choice models, supported by GIS data, to analyse the National Land Use Database, a register of more than 21,000 English brownfields—previously used sites with or without contamination that are currently unused or underused. Using spatial discrete choice models, including the first application of a spatial probit latent class model with class-specific neighbourhood effects, we find evidence of large local differences in the determinants of brownfields redevelopment in England and that the reuse decisions of adjacent sites affect the reuse of a site. We also find that sites with a history of industrial activities, large sites, and sites that are located in the poorest and bleakest areas of cities and regions of England are more difficult to redevelop. In particular, we find that the probability of reusing a brownfield increases by up to 8.5 % for a site privately owned compared to a site publicly owned and between 15 and 30 % if a site is located in London compared to the North West of England. We suggest that local tailored policies are more suitable than regional or national policies to boost the reuse of brownfield sites.

## Introduction

Urban sprawl and the development of greenfield sites that most developed countries are currently facing have recently pushed governments in several countries, including the EU, the US, Russia and China, to reuse previously developed land. The reuse of previously developed land can also improve the attractiveness of an area through the removal of neighbourhood eyesores, the clean-up of contamination if present, the creation of new jobs, new housing, or new commercial or industrial uses of the land, and, by keeping cities compact, can also conserve biodiversity and reduce energy consumption and greenhouse gas emissions (Alberini et al. 2005; Thornton et al. 2007; Williams 2012; Tang and Nathanail 2012; Wernstedt et al. 2013; Otsuka et al. 2013; Linn 2013; Dixon et al. 2006; Dixon and Adams 2008; Schulze Bäing and Wong 2012; Alberini 2007; Haninger et al. 2014).

There are more than 66,000 hectares of previously developed land, or brownfields in England, mostly located in the high-growth areas of greater London, the South East and East, that would be suitable to accommodate more than 200,000 new homes (DCLG 2015). The redevelopment of brownfields in England is, therefore, of major interest to planning agencies and developers.

In this paper we aim to investigate the determinants and the barriers of brownfields reuse in England using econometric techniques supported by Geographical Information Systems (GIS) data. Specifically, our study addresses English brownfields regeneration agenda with three related questions: (i) what (local) characteristics make a site more likely to be regenerated? (ii) has brownfields regeneration mostly occurred in city centres? (iii) should size and location specific policies be suggested to better tackle brownfields reuse?

Using data from the National Land Use Database for more than 21,000 brownfield sites, we explore the site characteristics that make a brownfield more likely to be regenerated. Specifically, we look at how the following variables have had an impact on the reuse of a site: previous use, site size, ownership type, whether the site is located in a city, a metropolis, or in rural areas, geographical location, and other geographical based variables, such as the population density and the index of deprivation of the area where the site is located, and the distance to the city centre. Our analysis aims at providing policy makers with indications to what has limited brownfields regeneration and what has favoured the reuse of previously developed sites in England. Our approach, based on observed data on the revealed preferences of local authorities’ brownfields regeneration projects, sheds lights on the successes and limitations of brownfields regeneration in England. Results from this analysis can provide guidance on where the government should act to lower barriers for brownfields regeneration.

Our analysis explores the effect of spatial unobserved variables affecting the decision to reuse brownfield sites by first using a spatial probit model that assumes the same spatial effect across all brownfields, and then by relaxing this assumption and allowing for different forms of unobserved spatial effects at local authority level using spatial random effects, spatial random parameters and spatial latent class models.

We find that, despite the apparent success achieved by the national brownfields policy most brownfields redevelopment has happened in “easy brownfields”. More resources, attention and specific policies are needed to redevelop “difficult brownfields”, such as large sites, sites that have previously been used for commercial and industrial activities, and sites that are located in the poorer and bleakest areas of cities and regions of England. Our spatial models find that brownfields reuse decisions are considerably affected by unobserved heterogeneity at local authority level, indicating that reuse decisions at brownfield sites should exploit the local specific characteristics of the areas where brownfields are located and therefore support the existence of local planning agencies responsible for driving the reuse of brownfields at local authority level, rather than having a national regeneration agency overlooking the reuse of brownfields.

The remaining of the paper is structured as follows: section two provides an overview of the brownfields regeneration policy, with a particular focus on England; section three reviews the literature on the barriers and drivers of brownfields redevelopment; section four describes the English dataset of previously developed land; section five presents the economic and econometric models; section six reports the results of the analysis; section seven concludes the paper with a discussion and some policy considerations.

## Brownfields Regeneration in England

In England, a brownfield is a previously used land—other than for agriculture—which is currently underused or unused (DCLG 2006), due to the presence of one or more factors, which may include contamination, affecting its reuse (English Partnerships 2006). Brownfield land is primarily the result of deindustrialization and suburbanization (Alker et al. 2000; Tang and Nathanail 2012), “it includes both vacant and derelict land and land currently in use with known potential for redevelopment. It excludes land that was previously developed where the remains have blended into the landscape over time” (ODPM 2005, p. 7). English brownfields do not necessarily have a history of contamination.

In England, the reuse of brownfields has been led by four political drivers to provide most new housing on previously developed land: (i) the high population density of England; (ii) the low population density of English cities, compared to average European densities; (iii) the large quantity of under-utilized land within urban areas; and, (iv) the population growth that will require more than two million new dwellings by the end of year 2020 (English Partnerships 2003).

When the “New” Labour Government came to power in 1997, it aimed at revitalising English urban centres through an “urban renaissance” and by building at least 60 % of new houses on brownfields or through the conversion of existing buildings by 2008 (DETR 2000c). In 2003, the Government launched the Sustainable Communities Programme (SCP) (ODPM 2003), which set up the goal of developing a new National Brownfield Strategy through the national regeneration agency, English Partnerships, and the Department for Communities and Local Government (DCLG 2009).

The initial results of the National Brownfields Strategy showed that, in 2005, 73 % of new dwellings were built on brownfields, but only 62 % of land for new housing was previously developed, mainly because usually urban houses are built at higher densities than those on pristine sites (Williams 2012). These considerations led the Coalition Government to implement major changes to brownfield development policy in 2010 by abolishing all existing regional housing and brownfield targets and make local planning authorities responsible for establishing the level and location of housing provision for the local area (Schulze Bäing and Wong 2012).

Information on brownfields is collected in the National Land Use Database (7), which comprises records of parcels of vacant and derelict land and buildings as well as those currently in use with potential for redevelopment. Contamination, unfortunately, is not recorded in the NLUD. Land contamination is dealt with in Part 2A of the Environmental Protection Act (EPA), which came into force in England in 2000 and aims at identifying and regulating the remediation of contaminated land that causes significant harm to human health or the environment or where there is a significant possibility of such harm to happen. Under this regulation, local authorities have to produce strategies through their planning system for inspecting their area for contaminated land and for overseeing the remediation of contamination (Otsuka et al. 2013). In addition, the European Union’s Environmental Liability Directive (2004/35/EC), transposed into English law through the Environmental Damage (Prevention and Remediation) Regulations in 2009 (SI 2009/153), requires the operator of a site where the environmental damage takes place to clean up any contamination caused by their activities.

## Literature Review

Several studies have looked at the barriers and drivers of brownfields regeneration. Most of this literature originates from the U.S., where a brownfield is any “real property, the expansion, redevelopment, or reuse of which may be complicated by the presence or potential presence of a hazardous substance, pollutant, or contaminant” (Small Business Liability Relief and Brownfields Revitalization Act, 2002).^1^ Although the U.S. definition requires the presence or potential presence of contamination, it excludes heavily contaminated sites, as those sites either have to be listed or proposed to be listed on the National Priorities List, or are remediated under the Toxic Substances Control Act 1976. This means that underused or unused sites that are heavily contaminated, such as Superfund sites, or have no presence and no potential presence of contamination are not classified as brownfields in the U.S., whilst they are in England. The English definition of brownfields is, therefore, much more extensive than the U.S. definition, as it includes previously developed land—with or without any level of contamination—that is currently underused or unused.

Most of the studies investigating the reuse of brownfields have focussed on the U.S. experience, and, in particular, on the effectiveness of voluntary remediation programs (Bartsch and Collaton 1997; Dennison 1998; Eisen 1999; Meyer and Lyons 2000; Wernstedt 2004; Wernstedt et al. 2006a, b, 2013; De Sousa 2003, 2004; De Sousa et al. 2009; Greenberg et al. 2001; Schoenbaum 2002; Page and Berger 2006; Alberini 2007; Schwarz et al. 2009; Guignet and Alberini 2010; Chilton et al. 2009; Blackman et al. 2010; Linn 2013; Haninger et al. 2014).

Alberini (2007) analyses the determinants of participation in voluntary remediation programs in Colorado using a probit model on 432 brownfields. She finds that the main determinants for participation are the size of the site and the surrounding land use. Using a hedonic price model, she further finds that properties with confirmed contamination sell at a 43–56 % discount and that participation in voluntary remediation results in a partial to complete price recovery. Guignet and Alberini (2010) find that participation in voluntary remediation programs in Baltimore, Maryland, is more likely for larger, less capital intensive sites, and industrial sites located in industrial areas, rather than heavily built sites close to residential areas.

Blackman et al. (2010) use multinomial logit models to find that participation in voluntary remediation programs in Oregon attracted both heavily contaminated sites and less contaminated ones.

Page and Berger (2006) discern several differences when comparing brownfields in Texas and in New York. They find that Texas has a higher percentage of sites with prior and current industrial uses than New York, whilst New York brownfields are more likely to be abandoned or vacant at the time they enter the voluntary remediation program. Most of the Texas sites are in urban areas and in central cities. Whilst industrial uses account for most of the properties enrolled in both states’ programs, suburban properties are more common in the New York program. They also find that sites that participate in voluntary remediation programs are on average smaller in New York than in Texas.

Schwarz et al. (2009) use California data to compare residential redevelopment for heavily contaminated sites subject to mandatory clean-up, Superfund sites, and less contaminated sites, eligible for voluntary remediation based on a risk based approach. They find less residential redevelopment at voluntary remediation sites compared to mandatory remediation sites, but also that sites with a higher probability of contamination are less likely to be redeveloped residentially, and more likely to be redeveloped industrially. They conclude that voluntary remediation programs based on a risk based approach are not well suited for boosting residential reuse of brownfields.

Linn (2013) uses a hedonic price model to study the effect of liability relief and brownfields redevelopment on the value of nearby properties in Cook County, Illinois, including the city of Chicago. After accounting for unobserved and time-varying variables that may be correlated with a certificate of liability relief, Linn finds that if a brownfield enters the Site Remediation Programme in Illinois and is certified, the value of a property 0.25 miles away increases by about 1 %, compared to an otherwise identical property that is not affected by the entry and certification.

Haninger et al. (2014) investigate the effect of the U.S. Environmental Protection Agency Brownfields Program at federal level and find that sites that have been remediated are associated with an increase of about 4.9–11.1 % in surrounding property values.

In England, brownfields regeneration has been studied mostly by planners, geographers and engineers (Adams and Watkins 2002; BURA 2006; Syms 2004; Roberts and Sykes 2000; Urban Task Force 1999, 2005; Diamond and Liddle 2005; Dair and Williams 2006; Harrison and Davies 2002; Dixon et al. 2006; Dixon 2007, 2006; Dixon et al. 2011; Bardos et al. 2000; Adams 2004; Cozens et al. 1999; Adams et al. 2001, 2010; Tang and Nathanail 2012; Otsuka et al. 2013; Williams 2012; Dixon and Adams 2008; Thornton et al. 2007; Schulze Bäing and Wong 2012; the European projects BERI, CLARINET, CABERNET, RESCUE). Most of these studies have used qualitative data and in-depth case studies, whilst studies using quantitative data to derive policy recommendations for brownfields reuse in England are less common.

Dixon et al. (2006) administered a mail survey to 987 commercial and residential property developers underpinned by structured interviews with eleven developers. They find that financial and other incentives given by the government were the main drivers for brownfields development.

Tang and Nathanail (2012), using ANOVA, analyse the NLUD dataset of brownfields in England and find that local authorities with higher percentages of derelict or vacant land are located in deprived areas, i.e. areas that fare badly in terms of income, employment, health, education, housing, crime and living environment. They also find that the increased density of housing on brownfields did not significantly reduce socio-economic deprivation at local authority^2^ level. Williams (2012) also examines the NLUD database and finds that derelict and vacant sites are mainly located in the industrial areas of the Midlands and Northern Regions of England, which are areas often affected by contamination that lack infrastructure and are usually not economical to develop. English Partnerships (2008), using GIS analysis of the NLUD dataset, find a strong correlation between brownfields and socioeconomic deprivation to conclude that more than 20 % of brownfields are located inside the 10 % most deprived areas. Analysis on the same dataset by Schulze Bäing (2010) further finds that brownfields reuse in most deprived areas has occurred at levels comparable to least deprived areas only in particular buoyant property market conditions in England during the 2005/6 period. Schulze Bäing and Wong (2012) use ANOVA to analyse the effect of economic indicators and deprivation indexes on brownfields regeneration using the NLUD dataset and find that high levels of brownfields reuse has boosted the real estate market for apartments in the most deprived areas and improved socioeconomic indicators in those areas.

## The National Land Use Database

This paper uses the data from the National Land Use Database (NLUD), which was created after the Government issued the policy document ‘Planning for the Communities for the Future’ (ODPM 1998). The NLUD initiative was a partnership project between Communities and Local Government, English Partnerships, the Improvement and Development Agency and Ordnance Survey. The database was created by the need to monitor the supply of brownfields to provide an adequate and strategic supply of land and buildings for housing and other economic activities. Data were provided on a yearly basis by local planning authorities that would collect information, such as geographical location, address, land use and planning attributes for vacant and derelict sites and other previously developed land and buildings that might have been available for redevelopment in England. The format of the data has changed during the years to keep the database consistent with the changes in the legislation. In addition, in (2007), English Partnerships became part of the Homes and Communities Agency, the National Housing and Regeneration Agency. This makes it difficult to compare database entries across years, and we therefore limit our analysis to the year 2006 dataset.

Five land types are collected within the NLUD: (i) previously developed land that is now vacant; (ii) vacant buildings; (iii) derelict land and buildings; (iv) land or buildings currently in use and allocated in the local plan and/or having planning permission; and, (v) land or buildings currently in use where it is known there is potential for redevelopment (but the sites do not have any plan allocation or planning permission) (NLUD 2000, 2003; ODPM 2006). Each site entry records the address and the British National Grid geographical reference, the previous and current activities (commercial, industrial, housing, or other), the area, the planning status, the proposed use, whether the site is suitable for housing, the most suitable use, an estimate of the housing density, and the ownership type, either public or private. Unfortunately the NLUD does not collect information on contamination at the sites. In fact, the NLUD and the regime for contaminated land for England (DETR 2000a, b) are separate and distinct exercises. The identification and classification of brownfields in the NLUD makes no representation on the likely presence of contamination. Some local authorities volunteer this information in the NLUD, but most do not. Therefore, it would be inappropriate to consider the NLUD as a registry of contaminated sites. Where sites are to be redeveloped, the planning and development control process ensures that any potential risks associated with contamination are properly identified and cleaned up.

As the NLUD sites are geo-referenced, we are able to augment the database with Geographical Information Systems (GIS) data obtained by the Office for National Statistics, Communities and Local Government. This augmentation includes information on: the population density of the wards^3^ where the sites are located, whether the site is located in a city, a metropolis or a rural area, the Index of Multiple Deprivation for 2004^4^ for the super output areas^5^ where sites are located, and the distances to the central business district.

## Modelling the Reuse of Previously Developed Land

Economic theory suggests that a brownfield will be reused if the net present value for a landowner from redeveloping the site is greater than the net present value of leaving it unused. The economic modelling approach adopted in this study further postulates that the regeneration of a brownfield is a function of the site characteristics (e.g. geographical location, size, distance to the central business district, previous activity at the site, housing suitability and ownership) and neighbourhood characteristics (e.g. population density and deprivation score of the area where the sites is located). Our hypothesis is that a site will be in use (*in_use*) if the net benefit to the owner,—defined here as profit—is greater than the profit derived from the site if it was unused (*unused*), including the option value arising from future costs, prices, policies, and the development of other nearby sites (Majd and Pindyck 1987; Wrenn et al. 2012). In accordance with Bockstael (1996), Irwin and Geoghegan (2001), Irwin and Bockstael (2004), Alberini (2007) and Guignet and Alberini (2010), the behavioral model is therefore, choose *in_use* over *unused* if and only if (iff):

1 $$\begin{aligned} \pi _{in\_use} >\pi _{unused} , \end{aligned}$$

where $\pi _{in\_use}$ and $\pi _{unused}$ are the true—but unobservable (i.e. latent)—profits associated with the site when it is in use and when it is unused respectively. So far we have assumed that variations in terms of development decisions are only due to variations in observable brownfield sites characteristics and surrounding neighbourhood features. However, in reality, landowners are heterogeneous and brownfield sites with same characteristics and same neighbourhood features may have different reuse decisions. For example, some landowners may be close to retirement and therefore unwilling to embark in a redevelopment project. Others might be more willing to redevelop a site for housing if they expect that a rapid demographic growth might increase the value of their land if redeveloped. These idiosyncrasies will create a distribution of unobservable factors, randomly distributed across the brownfield sites that will generate optimal reuse decisions conditional upon brownfield sites characteristics and neighbourhood features. Therefore, an owner will decide to reuse a site if the profit from reusing the site is higher than the profit from not using the site:

2 $$\begin{aligned}&\displaystyle \pi _{in\_use} >\pi _{unused} ,\nonumber \\&\displaystyle \quad \alpha _{in\_use} +{\varvec{\upbeta }}^{\prime }{} \mathbf{x}_{in\_use} +\varepsilon _{in\_use} >\alpha _{unused} +{\varvec{\upbeta }}^{\prime }\mathbf{x}_{unused} +\varepsilon _{unused} , \end{aligned}$$

where $\alpha$ is a constant term; ${\varvec{\upbeta }}$ is an unknown vector of parameters for the site and neighbourhood characteristics, $\mathbf{x}$; and, $\varepsilon$ is stochastic and is an unobservable factor of profit—and is treated as a random component. Due to the presence of this error component, the empirical model is driven by the probability that a site will be in use, i.e.:

3 $$\begin{aligned} Pr_{in\_use}= & {} Prob\left( {\pi _{in\_use} >\pi _{unused} ,\forall in\_use\ne unused} \right) ,\nonumber \\ Pr_{in\_use}= & {} Prob\left( {\alpha _{in\_use} +{\varvec{\upbeta }}^{\prime }{} \mathbf{x}_{in\_use} +\varepsilon _{in\_use} >{\varvec{\upbeta }}^{\prime }{} \mathbf{x}_{unused} +\varepsilon _{unused} ,\forall in\_use\ne unused} \right) ,\nonumber \\ Pr_{in\_use}= & {} Prob\left( {\varepsilon _{unused} -\varepsilon _{in\_use} <\alpha _{in\_use} +{\varvec{\upbeta }}^{\prime }\mathbf{x}_{in\_use} -{\varvec{\upbeta }}^{\prime }{} \mathbf{x}_{unused} ,\forall in\_use\ne unused} \right) \qquad \end{aligned}$$

Assuming the cumulative probability in Eq. (3) has a multivariate normal density leads to the ordinary probit model.

The estimation of a micro-scale spatial model requires a set of spatially articulated variables. The selection and specification of these variables is ideally determined by the factors expected to drive spatial variation in future profits. For example, heterogeneity in profits from reuse is related to landscape features such as land use and zoning requirements, accessibility, property tax rates and other variables that are unobservable to the researchers. In reality, the selection of explanatory variables is constrained by data availability. Augmenting the model with spatial variables, and exploring the effect of unobserved spatial heterogeneity, allows us to test whether location matters (Bella and Irwin 2002).

One may expect that the probability that a site is in use could affect the value of other, nearby properties, and also the decision to redevelop a surrounding brownfield site. To accommodate such possibility, and to account for the fact that omitted variable may be spatially correlated (Bockstael 1996), the probit model can be corrected to deal with spatial autocorrelation (McMillen 1992; Cho and Newman 2005). To capture these potential spatial dependencies, and to explore which model is more useful to derive policy recommendations in the analysis of brownfields reuse decisions, we use four different models.

We first use a spatial probit model based on the following profit function augmented with additional terms:

4 $$\begin{aligned} \pi _{in\_use} =\alpha _{in-use} +{\varvec{\upbeta }}^{\prime }\mathbf{x}_{in\_use} +\sum \nolimits _{s=1}^S \rho _s y_{s_{in-use} } +\varepsilon _{in-use}, \end{aligned}$$

where, $\rho$ defines a matrix of coefficients that define the influence that the planning decision in site $s = 1, 2,\ldots$. *S* has on the decision for a specific site, $s^{*}$. *S* is the number of sites that may potentially have an influence on the planning decision for the given site $s^{*}$ and $y_{s_{in\_use} }$ denotes the outcome at each site $s = 1, 2,\ldots$. *S* (set to unity if *in_use*, and zero otherwise). $\rho$ takes the form of a negative exponential function:

5 $$\begin{aligned} \rho _s =\lambda \hbox {exp}\left( {-\frac{D_s }{\gamma }} \right) , \end{aligned}$$

where, $$\lambda $$ and $$\gamma $$ are estimated parameters, and $$D_s $$ is the distance separating the two sites. A positive coefficient estimate for $$\lambda $$ implies the existence of a positive effect of planning decisions from adjacent sites to site $$s^{*}$$. That is, the decision to develop site $$s^{*}$$ is similar to the decision of development at sites nearby site $$s^{*}$$. The coefficient estimate for $$\gamma $$ captures part of the effect of the distance to adjacent sites to site $$s^{*}$$: a positive coefficient estimate for $$\gamma $$ suggests a positive and decreasing effect with distance from adjacent sites, whilst a negative coefficient would indicate a positive and increasing effect with distance from adjacent sites.

Implicit in this straightforward spatial probit specification is the assumption of homogeneity, which implies that the factors that determine whether or not a site is developed are the same across all sites. Notwithstanding the spatial autocorrelation already captured, it is possible that there may be differences in sites located in different local authorities—due to factors such as different political drivers or legislation and budget constraints that are not explicitly specified in the model. This “unobserved” heterogeneity suggests that observations within a local authority can be correlated by more than just space. Therefore, following the large and growing literature within discrete choice analysis that addresses unobserved heterogeneity (e.g., Campbell et al. 2014; Campbell and Erdem 2015), we capture this additional tier of possible correlation for sites located within the same local authority using a spatial random effects probit model, a spatial random parameters probit model, and a spatial latent class probit model. Indeed, models that account for unobserved heterogeneity have become standard practice in the analysis of discrete choices.

Under the spatial random effects probit model, we test the assumption that the status of sites in a particular local authority is useful information in predicting the status for other sites in the same local authority area and also in other local authority areas. This formulation assumes that the same random effects apply to all observations within the same local authority, but that they differ to observations outside the local authority.

We proceed to examine explicitly the unobserved heterogeneity across sites. This is achieved by portioning additively the stochastic component of profit into two parts:

6 $$\begin{aligned} \pi _{in\_use} =\alpha _{in-use} +{\varvec{\upbeta }}^{\prime }\mathbf{x}_{in\_use} +\sum \limits _{s=1}^S \rho _s y_{s_{in\_use} } +\left[ {{\varvec{\upeta }}_{in\_use} +\varepsilon _{in\_use} } \right] , \end{aligned}$$

where ${\varvec{\upeta }}$ is a vector of random terms. In the following section we present two models which use this form. The first of these models—labelled the spatial random parameters probit model—allows ${\varvec{\upeta }}$ to take an infinite set of values; whereas the second of these models—labelled the spatial latent class probit model—allows ${\varvec{\upeta }}$ to take finite set of distinct values. In both models the values of ${\varvec{\upeta }}$ can be either independent across sites or they can be the same for all sites within the same local authority. The spatial random parameters probit model and the spatial latent class probit model allow us to explore how brownfield reuse decisions are affected by the distribution of unobserved heterogeneity within local authorities, and therefore suggest whether local planning regeneration agencies might be better suited than a national planning regeneration agency to tackle brownfields reuse. To explain, the spatial random parameters probit model allows for the explanatory variables to take on a continuous distribution of values. The spatial latent class probit model restricts the possible values that the explanatory variables may take on to a finite number of parameters.

**Table 1:** Descriptive statistics

Variable	Acronym	Mean	Median	SD	Max	Min
AREA (hectares)		2.1	0.43	11.35	682.6	0.002
Population density. Persons per hectare in Ward in 2001	POP_DENS	24.47	24.47	21.99	236.05	0.04
Index of Multiple Deprivation score for super output areas for year 2004 where sites are located$^\mathrm{a}$	IMD_SCOR	29.7	24.57	19.95	86.36	1.16
Distance to the Central Business District (CBD) in km		1.644	0.871	2.158	27.535	0.007

Variable	Acronym	Mean	SD			
Site is in use, has been regenerated (dummy)	IN_USE	0.4091	0.4916			
Previous activity at the site was housing (dummy)	EX_HOU	0.1809	0.3849			
Previous activity at the site was commercial (dummy)	EX_COM	0.2002	0.4001			
Previous activity at the site was industrial (dummy)	EX_IND	0.2684	0.4431			
Previous activity at the site was agricultural (dummy)	EX_AGRIC	0.0085	0.0917			
Previous activity at the site was recreational area (dummy)	EX_REC	0.0093	0.0958			
Previous activity at the site was derelict (dummy)	EX_DER	0.0260	0.1590			
Previous activity at the site was unused (dummy)	EX_UNUSE	0.0070	0.0832			
Previous activity at the site was vacant building (dummy)	EX_VAC_B	0.0226	0.1486			
Previous activity at the site was vacant land (dummy)	EX_VAC_L	0.0718	0.2581			
Previous activity at the site was unknown (dummy)	EX_DK	0.2054	0.4040			
Site area is smaller than the median site area (0.43 hectares) (dummy)	SMALL	0.5018	0.500			
Site area is between 0.43 and 1.21 hectares (dummy)	MEDIUM	0.2491	0.2491			
Site area is larger than 1.21 hectares (dummy)	LARGE	0.2491	0.2491			
Housing suitability (dummy)	HOUSE_SU	0.6545	0.4755			
Privately owned (dummy)	PRIVATE	0.6099	0.4877			
Site is located in a city (dummy)	CITY	0.2915	0.4544			
Site is located in a metropolis (dummy)	METROPOL	0.2566	0.4367			
Site is located in a rural area (dummy)	RURAL	0.4519	0.4976			
Site is within 0.871 km from CBD (dummy)	DIST_50	0.5000	0.500			
Site is between 0.871 km and 1.85656 km from the CBD (dummy)	DIST_75	0.2500	0.4330			
Site is beyond 1.85656 km from CBD (dummy)	DIST_100	0.2500	0.4330			
East Midlands (dummy)	EASTMIDL	0.0962	0.2949			
East of England (dummy)	EASTENGL	0.0824	0.2749			
London (dummy)	LONDON	0.0542	0.2265			
North East (dummy)	NE	0.0675	0.2508			
North West (dummy)	NW	0.2169	0.4121			
South East (dummy)	SE	0.1323	0.3388			
South West (dummy)	SW	0.1042	0.3055			
West Midlands (dummy)	WESTMIDL	0.1329	0.3394			
Yorkshire and Humberside (dummy)	YORK_HUM	0.1134	0.317			

Number of observations is 21,808 for all variables; no missing values

$^\mathrm{a}$ Index was constructed by combining seven domain scores: Income, Employment, Health Deprivation and Disability, Education, Skills and Training, Barriers to Housing and Services, Crime, Living Environment. The higher the score the more deprived the super output area (ODPM 2004)

## Results

### Descriptive Statistics

Table 1 reports the descriptive statistics for the 21,808 brownfields recorded in the NLUD for 2006. Roughly 40 % of sites are currently in use. Industrial, commercial and residential activities are the main previous uses at the sites. The remaining previous uses are: recreational, agricultural, vacant buildings and land, unused and derelict. Local authorities do not know the previous activity at roughly 20 % of sites, most likely because these sites have been unused for a very long time and it is therefore difficult to gain information on the previous use at the site. The average (median) site is 2.1 (0.43) hectares, and is about 1.6 km (0.87 km) from the closest central business district. Most sites are located in cities, as 29.15 % are urban sites and 25.66 % are located in metropolis. More than 60 % of the sites are privately owned and deemed suitable for housing, one of the most pressing objectives of government planning policies. The average population density in the ward where sites are located is of about 24 persons per hectare. Finally, the super output area where sites are located has an average score of 29.7 for the 2004 Index of Multiple Deprivation (IMD). The Waverley Borough Council, County Surrey in the South East, represents the least deprived super output area, with an IMD score of 1.16, whilst Liverpool in the North West represents the most deprived super output area, with an IMD Score of 86.36.

**Fig. 1:**
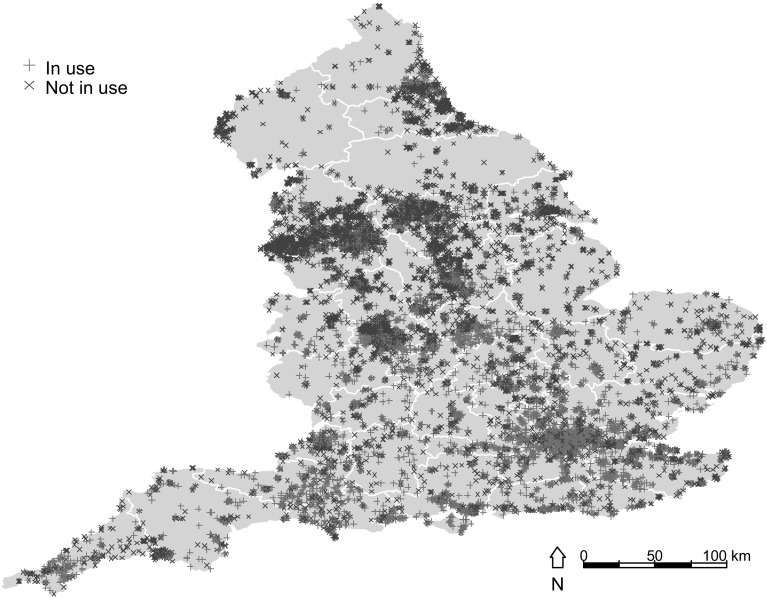
Brownfield sites in England

Figure 1 shows the English brownfields divided into two groups of sites currently in use, denoted using a (green) plus symbol, and unused, represented with a (red) cross symbol. Most brownfields are located in more densely populated areas, such as the capital, London, and other major (industrial) cities: Liverpool, Manchester, Hull, Newcastle Upon Tyne, Birmingham, Leeds, Plymouth, Portsmouth, Sheffield, Kirklees, St. Helens, Stoke-on-Trent, Swale, Tunbridge, Wells, Walsall, Wirral, and Wolverhampton. The map further shows a higher propensity of sites in use in the wealthy and more densely populated areas of the South and South East, compared to the poorer and less densely populated areas of the northern regions.

### The Determinants and Constraints of Brownfields Regeneration

Tables 2 and 3 present the results of our econometric models. Our analysis reflects the fact that sites belong to the same local authority, for a total of 353 local authorities, or groups. The tables also show that the spatial random effects and the spatial random parameters model^6^ improve the results obtained in the spatial probit model, indicating that specifications where the intrinsic correlation among sites within the same local authority is captured perform superiorly compared to specifications where observations are all assumed independent.^7^ The log likelihood function and the Akaike Information Criterion suggest that the three spatial models that allow for spatial unobserved heterogeneity at local authority level outperform the spatial probit model.

**Table 2:** Results: spatial econometric models

Variable	Random effects				Random parameters			
	Probit model		Probit model		Probit model			
	Coefficient	*t* statistic	Coefficient	*t* statistic	Means		Scale	
					Coefficient	*t* statistic	Coefficient	*t* statistic
Constant	3.4677	12.28	3.6283	6.54	4.0200	2.32	0.9681	7.28
EX_HOU	$-3.0189$	$-19.59$	$-3.5531$	$-14.36$	$-3.8375$	$-4.19$	0.6122	4.22
EX_COM	$-3.5776$	$-22.86$	$-4.1426$	$-16.25$	$-4.3772$	$-4.38$	0.3396	1.35
EX_IND	$-3.7091$	$-25.81$	$-4.3380$	$-17.60$	$-4.5466$	$-4.90$	0.0304	0.31
EX_AGRIC	$-3.0640$	$-8.99$	$-3.5505$	$-7.92$	$-3.0691$	$-2.42$	1.4845	1.33
EX_REC	$-3.1693$	$-16.84$	$-3.7759$	$-12.61$	$-4.0952$	$-3.80$	0.0703	0.15
EX_DER	$-4.8606$	$-20.57$	$-5.4703$	$-16.85$	$-6.2483$	$-3.64$	1.0712	1.62
EX_VAC_B	$-4.3241$	$-18.96$	$-4.8399$	$-13.60$	$-5.6177$	$-5.66$	1.4966	1.96
EX_VAC_L	$-4.6394$	$-19.75$	$-5.2037$	$-16.52$	$-6.7131$	$-5.04$	2.0075	4.84
EX_UNUSE	$-2.1402$	$-9.72$	$-2.8885$	$-9.88$	$-3.1437$	$-2.27$	0.7709	1.11
SMALL	0.0304	0.43	0.1530	2.57	0.1929	2.87	0.3125	5.05
MEDIUM	0.0053	0.12	$-0.0272$	$-0.56$	$-0.0263$	$-0.48$	0.1365	1.29
HOUSE_SU	0.4166	5.57	0.3531	4.61	0.4131	4.57	0.5770	6.42
PRIVATE	0.2364	3.97	0.3513	5.30	0.3366	3.32	0.4722	3.92
CITY	0.0164	0.22	0.0228	0.43	0.0185	0.32	0.0143	0.04
METROPOL	$-0.0185$	$-0.16$	0.0363	0.49	0.0558	0.40	0.0030	0.04
POP_DENS	$-0.0033$	$-1.56$	0.0000	0.02	0.0009	0.22	0.0031	1.79
IMD_SCOR	$-0.0180$	$-10.00$	$-0.0098$	$-6.50$	$-0.0102$	$-7.18$	0.0020	1.63
DIST_50	0.0868	1.22	$-0.0498$	$-0.79$	$-0.0487$	$-0.54$	0.0217	0.17
DIST_75	$-0.0845$	$-1.26$	$-0.1170$	$-1.99$	$-0.0759$	$-1.16$	0.0383	0.24
EASTMIDL	$-0.8596$	$-3.67$	$-0.7420$	$-1.32$	$-0.8179$	$-0.76$	0.2982	1.24
EASTENGL	$-0.7008$	$-3.12$	$-0.6358$	$-1.25$	$-0.6519$	$-0.60$	0.3201	1.06
NW	$-1.5282$	$-7.09$	$-1.5257$	$-3.25$	$-1.3767$	$-1.62$	0.1360	1.00
NE	$-1.0830$	$-4.66$	$-1.2785$	$-2.51$	$-1.5122$	$-1.58$	0.4833	2.46
SE	$-0.4961$	$-2.03$	$-0.1145$	$-0.26$	$-0.5946$	$-0.48$	0.0317	0.02
SW	$-0.4263$	$-1.79$	$-0.3356$	$-0.64$	$-0.9135$	$-0.89$	0.0048	0.04
WESTMIDL	$-0.7264$	$-3.35$	$-0.5656$	$-1.16$	$-0.4712$	$-0.55$	0.4302	2.81
YORK_HUM	$-1.4192$	$-4.96$	$-1.1118$	$-2.61$	$-0.6845$	$-0.90$	0.0107	0.03
lambda	0.0112	5.84	0.1853	4.10	0.1903	1.86		
gamma	11.2086	7.76	0.2403	4.46	0.2263	2.19		
Scale			0.8910	14.48				
Log likelihood function		$-6494.46$		$-5448.77$				$-5125.71$
McFadden Pseudo R-squared		0.568		0.637				0.657
Akaike Information Criterion		13,048.93		10,959.53				10,367.41
N		21,808		21,808				21,808

All *t* statistics are derived using local authority robust standard errors

**Table 3:** Spatial latent class model with 3 latent classes

Variable	Latent class/panel probit model					
	Class 1		Class 2		Class 3	
	Coefficient	*t* statistic	Coefficient	*t* statistic	Coefficient	*t* statistic
Constant	2.9965	9.40	3.1094	5.34	3.0806	5.10
EX_HOU	$-3.9650$	$-16.53$	$-2.2373$	$-4.32$	$-2.3706$	$-5.62$
EX_COM	$-4.2632$	$-18.00$	$-3.0270$	$-5.60$	$-3.1832$	$-7.96$
EX_IND	$-4.5379$	$-19.33$	$-3.2183$	$-6.27$	$-3.2929$	$-8.46$
EX_AGRIC	$-3.9167$	$-6.48$	$-1.9261$	$-1.76$	$-2.1507$	$-5.04$
EX_REC	$-3.8822$	$-11.48$	$-3.0354$	$-4.77$	$-2.3790$	$-4.76$
EX_DER	$-5.2846$	$-14.65$	$-3.5986$	$-3.52$	$-5.0131$	$-5.38$
EX_VAC_B	$-4.7763$	$-13.81$	$-4.0920$	$-5.86$	$-3.9181$	$-7.70$
EX_VAC_L	$-4.4502$	$-16.22$	$-5.5271$	$-8.98$	$-4.0852$	$-7.62$
EX_UNUSE	$-3.0196$	$-7.24$	$-2.0722$	$-2.87$	$-1.2495$	$-2.08$
SMALL	$-0.0961$	$-1.28$	0.2065	1.67	0.3719	3.09
MEDIUM	$-0.1119$	$-1.54$	$-0.1188$	$-1.16$	0.1286	1.27
HOUSE_SU	0.3089	2.52	0.6730	5.84	0.0715	0.50
PRIVATE	0.4514	5.51	$-0.2391$	$-2.33$	0.6349	4.54
CITY	$-0.1101$	$-1.18$	$-0.0247$	$-0.27$	0.0060	0.05
METROPOL	0.1583	1.53	0.1266	0.59	0.0996	0.57
POP_DENS	$-0.0013$	$-1.01$	$-0.0015$	$-0.66$	0.0074	2.30
IMD_SCOR	$-0.0114$	$-5.12$	$-0.0105$	$-2.91$	$-0.0048$	$-1.51$
DIST_50	0.0902	1.12	$-0.2321$	$-1.98$	0.0117	0.12
DIST_75	0.0066	0.08	$-0.2633$	$-2.24$	$-0.1866$	$-1.44$
EASTMIDL	$-1.0615$	$-3.60$	$-0.3013$	$-0.95$	$-1.6744$	$-3.72$
EASTENGL	$-0.3228$	$-1.51$	$-0.2719$	$-0.83$	$-1.0266$	$-2.20$
NW	$-1.2097$	$-5.99$	$-1.1256$	$-4.04$	$-1.8564$	$-4.23$
NE	$-0.6099$	$-2.84$	$-0.9188$	$-2.75$	$-0.5290$	$-1.04$
SE	$-0.4538$	$-1.88$	$-0.3936$	$-1.32$	$-0.2140$	$-0.49$
SW	$-0.0370$	$-0.15$	0.2796	0.93	$-0.9926$	$-2.24$
WESTMIDL	$-0.5289$	$-2.60$	$-0.7270$	$-2.24$	$-0.3761$	$-0.91$
YORK_HUM	$-1.4540$	$-3.59$	$-1.0711$	$-3.62$	$-1.7084$	$-3.30$
lambda	0.0136	11.59	0.2695	5.22	0.0167	6.15
gamma	16.8123	178.54	0.3356	6.06	14.7941	4.05
Probability	0.5075	0.25	0.2488	0.25	0.2437	0.25
Log likelihood function						$-5346.03$
McFadden Pseudo R-squared						0.640
Akaike Information Criterion						10,876.07
N						21,808

All *t* statistics are derived using local authority robust standard errors

The model that seems to explain better the data is the spatial random parameters probit model. This model was estimated specifying each of the variables as a normal distribution. The model was estimated using 100 Sobol draws. Focusing on the means attained from the spatial random parameters probit model, we find a number of interesting results. A site is more likely to be regenerated when local authorities do not have a clear information of the previous activity at the site (EX_DK is the reference dummy for previous activities at the site). In fact, all the dummy variables for the previous uses at the sites are negative and significant. Among these variables, EX_HOU, EX_AGRIC and EX_UNUSE have smaller coefficients, compared to the other previous uses, suggesting that when a site is used for residential, agricultural activities or was not previously used, it is more likely to be regenerated. This is a first important result that acknowledges the difficulties in developing sites that have been previously used for commercial and or industrial activities. These sites may in fact be considered more difficult to develop due to the presence of obsolete structures, and problems or fear of contamination. When we consider the size, we notice that smaller sites are more likely to be developed, being the coefficient of SMALL positive and significant, compared to MEDIUM and LARGE size sites (reference dummy).^8^

Our analysis wanted to explore to what extent the goal of the government of redeveloping sites located within urban cores to limit urban sprawl has been achieved. To address this question we look at the mean coefficient of the two dummy variables CITY and METROPOL that consider whether a site is located in a city or in a metropolis, and the two dummies for the distance to the city centre DIST_50 and DIST_75, with DIST_100 being the reference dummy. These dummy variables measure whether a site is located within the median distance to the central business district, DIST_50, between the median distance and the third quartile distance to the central business district, DIST_75, and between the third quartile and the fourth quartile distance to the central business district, DIST_100. None of the coefficient estimates for CITY, METROPOL, DIST_50, andDIST_75 are found to be significant, indicating that, on average, there has not been any significant difference in the redevelopment of sites in rural versus urban areas.

Being a site owned by the private sector (PRIVATE) or being suitable for housing (HOUSE_SU) makes it more likely to be reused, on average. Census characteristics affecting the probability of redeveloping a site are well captured by the Index of Multiple Deprivation, that indicates that the more deprived a site is, the less likely to be redeveloped. The population density, ceteris paribus, does not seem to influence particular pressure on the redevelopment of brownfields. Finally, the dummy variables for the geographical regions show that sites located in London (the reference dummy), West Midlands, South West, South East and Yorkshire and Humbershire are more likely to be regenerated, on average, compared to sites located in other regions.

Turning our attention to the spreads of the random parameters, we generally find significant heterogeneity for all variables. Indeed, in several cases the standard deviations are of a larger magnitude compared to their respective means—implying that the influence of these variables on brownfields redevelopment are very different across English local authorities.

**Fig. 2:**
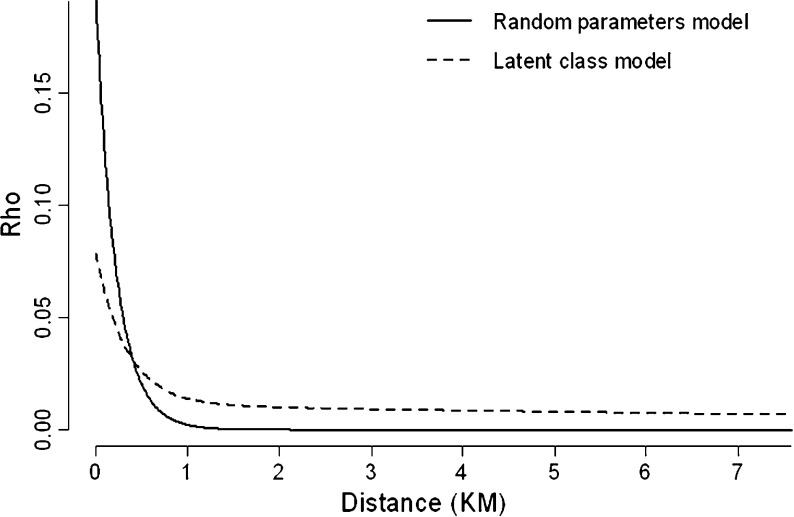
Distance decay curves for random parameters and latent class models

Also of interest are the results pertaining to the spatial dependency factor. Again, focusing on the random parameters model, we first remark that both parameters, lambda and gamma, are estimated with positive signs (as expected)—indicating the existence of similar redevelopment decisions in adjacent sites, and that closer sites have more influence on the development decision of a site. Furthermore, the fact that they are both significant means that accounting for this neighbourhood effect is necessary, and not accounting for spatial autocorrelation would lead to biased coefficient estimates. To assess the extent of this spatial effect, the spatial parameters can be used to predict a distance decay curve, as portrayed in Fig. 2. The curve produced from the random parameters model, suggests that brownfield redevelopments within a 1-km buffer have a direct impact on redevelopment.

Even though the spatial random parameters probit model is the model that fits our data better, the policy recommendations arising from its output are difficult to interpret, given the large heterogeneity captured by the spread of the random parameters. For this reason in the next subsection we investigate the results of a spatial latent class probit model.

### Geographical Differences in Brownfields Regeneration

**Fig. 3:**
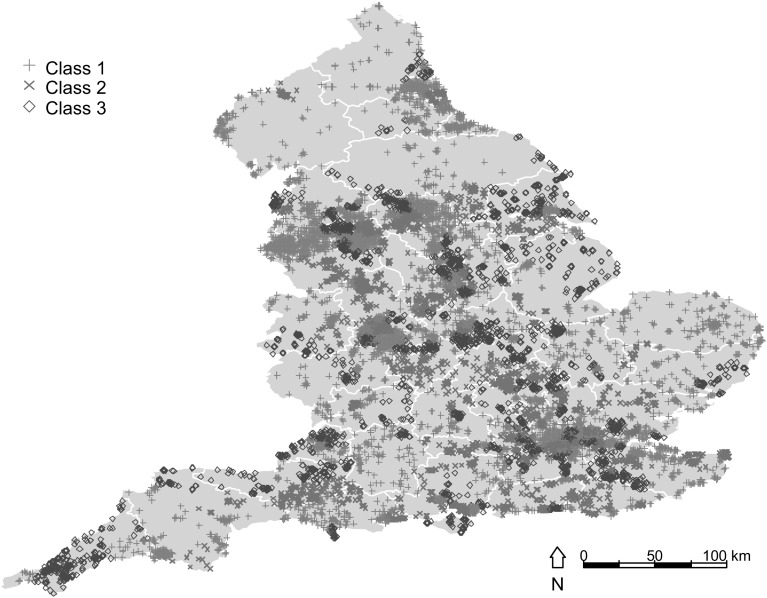
Distribution of brownfields sites according to conditional latent class probabilities

Table 3 reports the results of a spatial latent class probit model estimated with three latent classes. The three classes, representing three groups of local authorities, were chosen according to the model minimising various information criteria.^9^ At the bottom of the table, the estimated prior probabilities for each class show that our sites are about 51 % likely to belong to class 1, 25 % to class 2 and 24 % to class 3. Figure 3 reports a graphical representation of the brownfield sites in three classes, which we base on the conditional latent class probabilities. This model allows us to investigate better the relationship between brownfield reuse and location of the sites. Sites located in the South East, North East, and North West, are more likely to belong to class 1. Sites in the South West are more likely to belong to class 3, and sites in class 2 are quite evenly spread in the South and in the Centre of England. In London, sites are most likely to belong to class 1 or 2.

The spatial latent class probit model finds that brownfield sites whose previous activity was unknown are more likely to be redeveloped across all the three classes. This suggests that sites that appear to not have been used for a long time, everything else being equal, are more likely to be reused. Making comparisons across classes, we find that large sites are most likely to be redeveloped under class 1, whereas small sites are most likely to be redeveloped under classes 2 and 3. In contrast to classes 1 and 3, being a site owned by the private sector makes a sites significantly less likely to be redeveloped in class 2. In the case of class 3, local authorities with a higher population density have higher likelihood of brownfields redevelopment. Not surprisingly, given the results portrayed in Fig. 3, we find that the dummy variables for the geographical regions also differ across the classes. Interestingly, we find that the neighbourhood effects also differ across the classes. This is an important result. Whilst the existence of brownfield redevelopment in adjacent sites has a positive effect on all redevelopment, the spatial spillover is not the same in all local authorities. For example, the spatial effect of the influence of the development decision of a brownfield site is stronger in sites redeveloped in class 2, but this influence declines much more sharply for these sites, compared to sites redeveloped in classes 1 and 3, where the effect of nearby sites is weaker but spreads in a longer distance.

Empirical issues aside, to the best of our knowledge this is the first application to have estimated a spatial probit latent class model with class-specific neighbourhood effects. Therefore, this also represents an important methodological contribution. The size and the extent of the spatial effect retrieved from this model (weighted across the three latent classes) is shown in Fig. 2. From this, we can see a much larger buffer zone (perhaps as large as 8-km).

## Conclusions

This paper has looked at the determinants and barriers of brownfields redevelopment in England. We assumed that, in England, the development of brownfields—sites that may or may not have been contaminated, which have been previously used and are currently unused or underused—depends on the characteristics of the sites, location, previous use, current use, size, ownership type, socio-economic conditions of the people living in the areas where the brownfields are located, as well as unobserved characteristics of the local authority where brownfields are based, such as political drivers, legislation, planning policies, budgets, and the influence that the development of adjacent brownfields has on a decision of a site to be reused. We control for unobserved heterogeneity at local authority level using random coefficients, random parameters and latent class probit models, and use a correction for spatial autocorrelation to capture the effect that the development decision of surrounding brownfield sites have on the reuse decision of a site.

The results from the econometric models can be used to estimate the effect of a change in the Index of Multiple Deprivation where a brownfield is located or of a change in the characteristics of brownfields in the probability that a brownfield site is reused. Using the results from Table 3, considering a spatial effect of surrounding brownfields within 1 km from a brownfield site, we find that for a large, publicly owned, former industrial site located in London in an area with an Index of Multiple Deprivation equal to 80, reducing deprivation to a level of 20 increases the probability of redeveloping a brownfield site by 6.4 % for a site belonging to class 1, by 12.3 % for a site belonging to class 2, and by 4.5 % for a site belonging to class 3. If we further explore the effect of a change in ownership from public to private, we find that the probability of reusing a site increases by 8.5 % for a site belonging to class 1, it decreases by 3.7 % for a site belonging to class 2, and remains virtually unchanged for a site belonging to class 3. We can further explore the difference in probabilities of reuse of a brownfield site in different regions in England. If we consider two identical large former industrial sites, privately owned, located in an area with an index of Multiple Deprivation equal to 20, one in London and one in the North West, a region with a strong history of past industrial activities, we find that the probability of reusing a brownfield site in London is 15.3, 23.7, and 34.2 % higher than in the North West for sites belonging to class 1, class 2 and class 3 respectively. These results show that there are big differences in the effect that site characteristics have on brownfields reuse, indicating that the use of flexible models, such as spatial latent class and random parameters probit models that account for unobserved heterogeneity at local authority level and spatial autocorrelation provide invaluable tools for exploring the determinants of brownfields reuse.

The results highlight that the brownfield community has done some progress in redeveloping previously developed sites, but that some constraints still need to be overcome. The goals of the government of building most new houses on brownfields is being achieved, but more resources, attention and specific policies are needed to redevelop “difficult” sites, such as large sites, sites that have previously been used for commercial, or industrial activities, sites that are located in the poorer and bleakest areas of cities and regions of England. These might also be sites that suffer from the presence or suspected presence of contamination, as this is more likely in general to be found in industrial and commercial sites and larger sites. However, we cannot derive conclusions on the effect of contamination on brownfields redevelopment in England from this study, as the NLUD database does not collect information on contamination. This is unfortunate, as the dataset is otherwise rich in information. It would be desirable that in the future all local authorities released the information on contamination and clean-up on their brownfield sites in the NLUD.

It is finally interesting to highlight how the government does not seem to fully understand the opportunity cost of not developing publicly owned sites, as public ownership seems to be a constraint in regeneration for most brownfields. Perhaps, it is possible that privately owned sites, which are more likely to be redeveloped, might be more valuable sites than publicly owned sites, and hence encourage private owners to reuse them. Unfortunately, the NLUD does not include information on the value of the brownfield sites, and this is a second limitation of the dataset that we would urge local authorities to report in the future, so that researchers can investigate hedonic studies of brownfields redevelopment in England.

We have also found a strong unobserved heterogeneity in reuse decisions of brownfields, captured by the unobserved local authorities’ characteristics in the analysis, and a positive effect on reuse decisions from reuse decisions at surrounding brownfield sites. Our results, that show considerable differences in reuse decisions captured by unobserved heterogeneity at local authority level, therefore, support the recent direction of the government to make local planning authorities, rather than regional planning authorities, responsible for brownfields regeneration (Schulze Bäing and Wong 2012). Finally, we believe that the recommendations of the Barker Review (2006) to use policy instruments, such as introducing a charge on vacant and derelict brownfield land and a subsidy to help developers bring forward hard-to-remediate brownfield sites should still be pursued, but we also recommend that a specific set of policy instruments should be used to address publicly owned brownfields, which may be less profitable sites to develop. Finally, we should note that the dataset used in this analysis is about 10 years old. Unfortunately, more recent datasets of the NLUD are either not available, or do not have the same number of observations. For example, the 2012 dataset has got only 8860 observations, about one third of the dataset used in the current study. We would encourage the government to improve the collection of data of previously used land so that it will be possible in the future to conduct longitudinal studies to examine the reuse of brownfields.

## Footnotes

1 Federal and state legislation pertinent to U.S. brownfield policy is numerous and diverse. The most important includes the Resource Conservation and Recovery Act (RCRA), the Community Reinvestment Act (CRA), the Comprehensive Environmental Response, Compensation and Liability Act (CERCLA) or Superfund, and the Small Business Liability and Brownfields Revitalization Act. CERCLA, in particular, imposed joint and several liability on all “potentially responsible parties”, de facto making anyone with a proven link to a contaminated site potentially liable for the contamination at the site. This has led not only to the potentially responsible parties attempting to implicate one another for damages, but also greatly reduced the incentives to purchase or reuse a potentially contaminated site. In 2002, the Small Business Liability Relief and Brownfields Revitalization Act (i.e., the “Brownfields Law”) was signed as an amendment to CERCLA to provide more financial and technical assistance to brownfield remediation, including cleanup and assessment grants and liability protections, and to stimulate states-led voluntary cleanup programs.

2 Local authorities in England are responsible to provide services, such as education, social services, planning, waste management, trading standards, emergency planning, roads and transportation, housing, environmental health, parks, markets and fairs, to the population in their area. There are five different types of local authorities in England and they are divided into single-tier and two-tier authorities. Single tier authorities are: Metropolitan Authorities, London Boroughs, and Unitary or Shire Authorities. Two tier authorities are comprised of a County Council, and a District Council. There are about 400 local authorities in England.

3 Wards are the spatial units to elect councillors in England. The geographical size of a ward depends on the population density of an area. On average, a ward includes about 5500 people. There are 7669 wards in England.

4 The Index of Multiple Deprivation 2004 (ODPM 2004) is calculated by the Social Disadvantage Research Centre at the University of Oxford for super output areas lower layer in England. The index is constructed by combining seven transformed Domain Indexes, using the following weights: Income (22.5 %), Employment (22.5 %), Health Deprivation and Disability (13.5 %), Education, Skills and Training (13.5 %), Barriers to Housing and Services (9.3 %), Crime (9.3 %), Living Environment (9.3 %).

5 Super output areas are a set of geographical areas developed following the 2001 census, initially to facilitate the calculation of the Index of Multiple Deprivation 2004. The aim is to produce a set of areas of consistent size suitable for the publication of socioeconomic data related to small areas comprising a population of about 1000–3000 in each area.

6 Note that the choice probabilities in the spatial random effects and spatial random parameters model cannot be calculated exactly (because the integrals do not have a closed form). Instead, they were approximated through simulating the log-likelihood with 100 Sobol draws per local authority. To address the correlation among local authorities, the same random draws were used for all sites within the same local authority. For each random draw, the product of the simulated probabilities within each local authority is derived. The average of these products gives the contribution of the local authority to the likelihood function.

7 We also ran an ordinary probit model but found that the spatial probit model outperformed it. This result is also indicated by the spatial coefficient estimates of lambda and gamma in Table 2.

8 In specifications not reported here, we explored the use of continuous variables for the size of the areas of the brownfields, and for the distance to the city centre. For example, we used the inverse of the distance (Ihlanfeldt and Taylor 2004) or the logarithm of the distance (Longo and Alberini 2006), but we found the sign of the coefficient estimates not statistically significant.

9 We tested up to four latent classes. However, we observed that going beyond three classes reduced significance of parameter estimates since classes were associated with lower probabilities. We also remark that the sample-likelihood function was much more susceptible to local maxima and we were less confident of reaching a unique maximum. As was the case in the spatial random effects and spatial random parameters models we derived the products of predicted probabilities but this time using the class-specific parameters. These were then weighted according to the class membership parameters.
